# Screening Procedure for Hemihypertrophy: Preliminary Results of International Multicenter Prospective Study

**DOI:** 10.5195/cajgh.2019.336

**Published:** 2019-03-01

**Authors:** Michael Vaiman, Phillip Shilco, Yulia Roitblat, Nicolas Padilla-Raygoza, Aidan Leit, Aaron Kavin, Edan Schonberger, Liliia Nehuliaieva, Noa Buchris, Michael Shterenshis

**Affiliations:** 1 Department of Otolaryngology, Assaf Harofeh Medical Center, Affiliated with Sackler Faculty of Medicine, Tel Aviv University, Tel-Aviv, Israel; 2 “Briut HaShen” Dental Health Clinic, Jerusalem, Israel; 3 Department of Sciences, Belkind School for Special Education, Rishon-LeZion, Israel; 4 Department of Nursing and Obstetrics, Division of Health Sciences and Engineering, Campus Celaya-Salvatierra, University of Guanajuato, Mexico; 5 Department of Sciences, The Harley School, Rochester, NY, USA; 6 Department of Anatomy, Mount Moriah College, Sydney, Australia; 7 Department of Pediatrics, Danylo Halytsky Lviv National Medical University, Lviv, Ukraine; 8 Dept. of Sciences, El Camino Real Charter High School, Woodland Hills, CA, USA; 9 Science Research Department, Alexander Muss High School in Israel (AMHSI) affiliated with Alexander Muss Institute for Israel Education (AMIIE), Hod HaSharon, Israel

**Keywords:** Hemihypertrophy, Hemihyperplasia, Asymmetric regional body overgrowth, Body asymmetry, Adolescents

## Abstract

**Introduction:**

Isolated or congenital hemihypertrophy is a rare disorder characterized by asymmetric overgrowth of one side of the body. This article describes the protocol and preliminary results of a lateral body asymmetry (hemihypertrophy) screening procedure performed in healthy adolescents in a multicenter study. The reported incidence of hemihypertrophy varies between different publications and standardized protocols are needed to improve research in this area.

**Methods:**

Our screening program is taking place in Australia, Israel, Mexico, Ukraine and USA. Procedure includes two steps: (1) “three measurements – three questions” screening, or assessment of face, palms, and shins; (2) in-depth assessment of selected cases in order to exclude localized, lesional, and syndrome-related cases as well as body asymmetry within normative range and to select suspected cases of isolated hemihypertrophy. This step includes measurements of various anatomical regions and a detailed questionnaire.

**Results:**

At this stage, the screening procedure is completed and the selected participants are advised to refer to medical institutions for further clinical and genetic follow up to exclude possible tumors and other accompanying disorders.

**Conclusion:**

We present an easy-to-use selection tool to identify children with suspected IH, which results in the selection of the risk group that may benefit from referral to a pediatrician and a clinical geneticist.

## Introduction

Isolated or congenital hemihypertrophy (IH, isolated hemihyperplasia, Human Genetic Disorders Code: OMIM 23500; lateralized overgrowth) is a rare disorder characterized by asymmetric overgrowth of one side of the body. Statistics vary on how many people actually have this pathology because the incidence of IH has been reported to range from 1:13,000 to 1:86,000 live births.^1^ Such estimation may be inaccurate, as the age of onset of IH can vary between the cases. Numerous case reports describe patients who were first referred to a pediatrician between 10 and18 years of age.^2^–^4^

In general, patients with IH are at an increased risk for medullary sponge kidneys, arteriovenous abnormalities, and tumors in the abdomen. Therefore, having a standardized screening procedure for such cases is desirable.^5^,^6^ Adolescents are not usually at risk for tumor development, yet some internal abnormalities may accompany visible body asymmetry, which may severely affect quality of adolescents’ life.^1^,^2^,^7^ Diagnosing IH is complicated by similar conditions, including Beckwith-Wiedemann syndrome, Proteus syndrome, Klippel-Trenaunay syndrome, and Sotos syndrome, which have symptoms similar to IH. Most of these disorders are presented at birth or identified in early childhood, thus the main purpose of the screening is to detect IH in older children.

This study describes a screening procedure and the selection tool for adolescent IH cases. Recently published article by Mark et al. describes in detail how a practitioner should assess and investigate a patient with suspected IH and what surveillance strategy should be applied to such patients.^8^ Our screening procedure may help to select potential IH cases that may benefit from such fifteen-minute consultation. This article describes the protocol and preliminary results of a lateral body asymmetry (hemihypertrophy) screening procedure performed in healthy adolescents in a multicenter study.

## Screening procedure

### Study population

Our screening is currently taking place in several countries including USA, Australia, Israel, Mexico, and Ukraine, aiming to screen at least 5000 participants in each country. Institutional Review Board approvals were acquired for each respective institution that conducts this research. Inclusion criteria were the following: healthy individuals, aged 15–18, of both sexes. Exclusion criteria were the following: individuals with known disorders such as Beckwith-Wiedemann syndrome, mosaic trisomy 8, Proteus syndrome, Russell-Silver syndrome (hemihypotrophy), Klippel-Trenaunay syndrome, Sotos syndrome, neurofibromatosis Type 1, and Bannayan-Riley syndrome. Prospective participants were excluded from the study if their body asymmetry was a result of known trauma, lymphatic malformation/lymphedema, and vascular malformations.

### Initial screening

**Figure 1:**
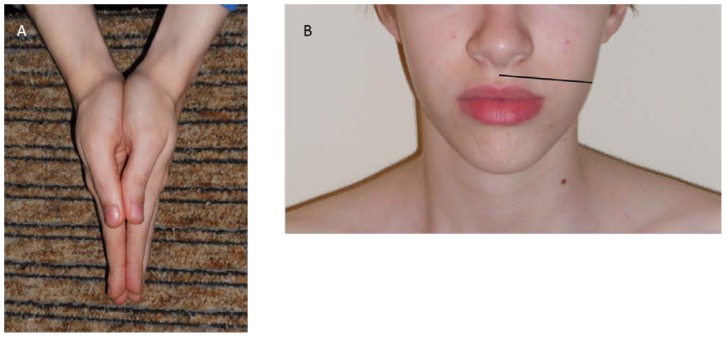
A) A test to compare the sizes of the palms; B) Assessing facial asymmetry. The distance between philtrum and the angle of a lower jaw is measured.

The screening procedure includes two steps. The initial step is “three measurements – three questions” screening, or “face – palms – shins survey”. The three measurements are:

1. The measurement of the fullest part of the calf below the knee with a difference of circumference >1.5 cm between the two legs reported as significant,
2. Comparison of palms (Figure 1a), with a difference of length >1 cm between the two hands reported as significant,
3. Assessing face asymmetry by measuring the distance between philtrum (below the nose) and the angle of a lower jaw (Figure 1b) with a difference of width >1.5 cm as significant.

Each participant is asked the following three questions:

1. Is he/she bothered with his/her body asymmetry?
2. Has he/she ever had dental braces, orthodontic treatment, lower jaw repositioning, etc.
3. Has he/she ever visited a podiatrist, had orthopedic treatment, or worn shoes of different sizes?

The total score for a participant after all measurements and answers ranges from 0 to 9. Each positive answer to the question adds 1 point; each asymmetrical measurement adds 1 point if the asymmetry does not psychologically bother a participant, and 2 points if the asymmetry is reported as bothersome to a participant. The selection for the second step is based on the assessment of the score: score 0 to 2 – definitely not selected, score 4 to 9 – definitely selected, score 3– selected if all three points were gained from the measurements data. About five minutes per participant are needed to complete this stage of assessment.

### Follow-up screening

The subsequent step is in-depth assessment of selected cases to select suspected cases of IH and exclude localized or lesional cases, in which initial findings are not supported by anatomical changes in other parts of the body or the history of trauma is present. This step includes measurements of various anatomical regions (length of the soles, legs and arms, the circumference of the thigh 10 cm above the upper edge of the patella, theleft and right half-circumferences from the navel to the spinous process of L4, presence/absence of scoliosis). Each additional asymmetrical measurement (>1.5 cm as significant) adds 1 point to the score. An additional question is asked: “Are there any other members of your family with any kind of body asymmetry?” A positive answer adds 3 points to the score. The subsequent questions concern the type of malignancy in the family (yes/no, which type, if yes – 1 point is added; if Wilms tumor (nephroblastoma that is usually diagnosed in children under the age of 6) – 3 points are added), and ultrasonography of the abdomen (performed: yes/no, if no - 1 point is added). If three or more points are added to the initial score that a participant obtained at the first stage, the participant is assigned to the risk group. About 10–15 minutes per participant are needed for these measurements and questions.

At this stage, the screening procedure ends and the selected participants, “the risk group”, are advised to refer to medical institutions for further investigations to confirm/disprove IH, for differential diagnosis between hemihypertrophy and hemihypotrophy, that may involve further investigations, and to exclude possible accompanying disorders.

## Results and Discussion

The preliminary findings as for December 2018 are presented in Table 1. These findings indicate the incidence of IH as 1:5000 that is significantly higher than previous 1:13,000 to 1:86,000 estimates. While the initial genetic mutation that leads to IH and the subsequent syndromes appeared in Europe in the 19^th^ century, it was rapidly spreading over the planet in the20^th^ century and became a global phenomenon in the 21^st^ century.^1^,^4^,^6^–^8^

The above-described screening procedure is taking place using the protocol described above in several countries across various continents. We would like to suggest that a higher number of children and adolescents with suspected IH need to be referred to pediatricians for consultation to confirm or rule out the disorder in order to improve quality of life of the patients and to ensure that no internal anatomical changes, such as possible accompanying hyperplasia/hypertrophy of the abdominal organs, are evolving. Recently published preliminary results based on 6000 participants^9^ estimate the incidence of IH ranging from 1:13,000 to 1:86,000^1^ is an underestimation. Our goal, therefore, is to screen 25,000 participants internationally to estimate global prevalence of the disorder. Our preliminary assessment corroborates the findings of Schook et al.^10^ These authors investigated 170 children with a referral diagnosis of lower extremity lymphedema. They confirmed this diagnosis in only 72.9 percent of patients and found IH in 8.7 percent of the initial cohort. These important findings indicate that IH can be overlooked or misdiagnosed which may lead to incorrect management of some patients.

The rationale for selecting the first step measurements was based on the fact that IH most often reveals itself through facial hemihypertrophy and hypertrophy of the limbs. The 1.5 cm difference was chosen as significant because most study participants had left-right face and shins asymmetry between 0.5 and 1.5 cm.^9^ The length of the palms, however, was symmetrical in most of the cases. The first question was chosen to assess the quality of life and psychological issues. The second question probes further into the facial asymmetry. Misdevelopment of the lower jaw is a significant indicator of IH. IH is associated with dental and oral abnormalities, including dental arch asymmetry and differences of dental development in the right and left jaws.^11^,^12^ Maxillo-facial surgeons may approach the case as an isolated pathology and IH can be overlooked. The same is true for the feet. For abnormal limb size, orthopedic treatment and corrective shoes can be recommended as palliative measures, but podiatrists may overlook IH.

The subsequent steps will aim to collect more data within the survey frame. While numerous reports on genetic abnormalities in IH cases were published, additional assessment of other family members to rule out any kind of body asymmetry may be highly informative because hereditary basis of the disorder, the instability of the 11p15.5 chromosomal region, is very well established.^1^–^5^,^8^–^10^ A close connection between IH and Wilms tumor (nephroblastoma, a common pediatric neoplasm of the kidney) was postulated already in the 1950s and was confirmed in more recent publications.^13^,^14^ While the overall median age for Wilms tumor is 3.5 years, adolescents and even adults also can be affected.^15^,^16^ That is why the question about Wilms tumor is one of the key questions in the questionnaire, despite the fact that tumor risk for adolescents is low.

We present an easy-to-use selection tool to identify subjects with suspected IH who may benefit from further clinical investigation and genetic counseling. The selection tool involves anthropological measurements and questions concerning family history, quality of life, and details of the lateral body asymmetry, which results in the selection of the risk group that may benefit from referral to a pediatrician and a clinical geneticist.

## Figures and Tables

**Table 1:** Preliminary results of the research (for December 2018) country by country included in the survey.

Country	Goal	# of participants Screened	Selected for Stage 2	Selected for “Risk group”	Diagnosis was confirmed
Australia	5000	478	2	1	0
Israel	5000	5000	185	41	1
Mexico	5000	370	0	0	0
Ukraine	5000	1834	4	2	0
USA	5000	2384	76	29	1

Total	25,000	10,066	267	73	2
